# Chinese women’s years of education and subjective well-being: an empirical analysis based on ordered Logit model and coupling coordination model

**DOI:** 10.3389/fpsyg.2024.1341995

**Published:** 2024-09-18

**Authors:** Ting Qin, Pingqiang Wei, Chengyi Zhu

**Affiliations:** ^1^School of Literature and Journalism, Xihua University, Chengdu, China; ^2^Xihua University Yibin Branch Management Committee, Yibin, China; ^3^School of Computer and Software Engineering, Xihua University, Chengdu, China

**Keywords:** subjective well-being, years of education, sex differences, Logit model, coupling coordination model, CGSS, authentic proof analysis

## Abstract

In modern society, the improvement of women’s education level has become one of the important indicators of national development and social progress. Although there are many useful explorations on the relationship between education and subjective well-being, the research on women’s years of education and subjective well-being is very limited. The article focuses on women’s years of education to determine whether and how to affect subjective well-being. This study is based on the China general social survey in 2021. The ordered Logit model was used to analyze the impact of women’s years of education on subjective well-being, and a binary coupling coordination model was constructed to test the above two variables. The results show that the longer the education years of women, the stronger the subjective well-being. The benchmark regression results show that women’s years of education have positive and negative effects on subjective well-being through economic status, physical and mental health, ecological environment, social cognition and personal cognition. The analysis of coupling coordination degree shows that the coupling between the years of education and subjective well-being of women in coastal areas and economically developed areas is the strongest, and the subjective well-being is better realized by increasing the years of education. Based on the above research results, this paper provides some practical suggestions for improving women’s subjective well-being, and provides some valuable references for women to effectively balance husband-wife relationship, family relationship and work relationship, improve women’s years of education and better obtain happiness.

## 1 Introduction

The pursuit of free and comprehensive development and the realization of a happy life are the eternal goals of mankind. Are the people satisfied? Is the people happy? As an important indicator of government work, governments at all levels in the world regard improving people’s well-being and enhancing people’s subjective well-being as the fundamental goal of development. The “World Happiness Index Report, 2022” released by the United Nations points out that China’s happiness ranks 72nd in the world, and the overall happiness of the country has improved, but there is still much room for improvement. There are many factors affecting happiness, and the improvement of Chinese residents’ happiness has a long way to go. With the expansion of college enrollment in mainland China, in order to better and effectively reduce the burden of heavy homework and off-campus training for students in compulsory education, the Chinese government promulgated the “Opinions on Further Reducing the Burden of Students Homework and Off-campus Training in Compulsory Education” (referred to as the “Double Reduction” policy) in July 2021. With the popularization of higher education and the impact of the compulsory education market economy, the voice of “useless education” has occasionally occurred, and people have questioned the improvement of academic qualifications to enhance subjective well-being. With the progress of society and the continuous development of economy, the academic community has conducted in-depth research on the “happiness paradox.” The effect of education level on happiness experience through income has gradually weakened (Tang, 2019; Li T. et al., 2023; Li X. et al., 2023), and once again questioned that the improvement of academic qualifications does not necessarily lead to a stronger sense of happiness. To this end, we need to clarify whether and how years of education affect subjective well-being.

China has a vast geographical area and a large population, and there is a large gap in happiness perception between regions and different groups of people. The “People’s Happiness Index Research Report, 2022” released by Tsinghua University points out that although China’s national happiness index has continued to improve since the reform and opening up, regional differences are very obvious. Scholars believe that education level (Huang et al., 2017, 2024), wage income (Zhou and Huang, 2018), gender, age, marital status (Wei et al., 2020), material and non-material acquisition (Qu and Liu, 2022) are the main factors affecting subjective well-being. The above research mainly takes a single factor as the research object, or the final conclusion does not involve the influence of years of education between gender differences on subjective well-being. There is still a lack of systematic quantitative research on the complex relationship between women’s years of education and subjective well-being. Under the specific cultural background of China, the mechanism of what factors interweave to affect women’s subjective well-being still needs to be further explored. The purpose of this study is to fill this research gap by systematically investigating and analyzing the relationship between women’s years of education and subjective well-being.

## 2 Literature review and research hypothesis

### 2.1 Subjective well-being

Social progress includes both economic and social well-being (Easterlin, 1974). It shows that both economic well-being and social well-being are sources of subjective well-being. Subjective well-being is the personal perception and experience of positive and negative emotions, as well as the specific cognition and evaluation of life satisfaction (Diener et al., 2002). This is a more comprehensive definition of subjective well-being, which is not only a response to emotions, but also a response to life, and then discusses its evaluation basis and influencing factors. There are many factors affecting subjective well-being. The scholars mainly discuss: income level (Luo, 2006), health status (Cattaneo et al., 2009), environmental status (Levinson, 2012), gender difference (Wei et al., 2020), sports diet (Yildizhan and Yazici, 2020), housing status (Liu et al., 2022), marital status (Hu et al., 2022), income gap (Liu and Zhou, 2023), education level (Chen and Zhang, 2023; Yang and Du, 2023), immigration (Zunino, 2021), digital literacy effect (Wang et al., 2022). On this basis, this study deeply studies the influence of women’s years of education on subjective well-being.

### 2.2 Years of education and subjective well-being

Hadjar et al. (2008) proved that education is robust to life satisfaction. Xu et al. (2022) proved that education will affect the trust of rural women and encourage women to receive a higher level of education. Bien and Bien-Barkowska (2016), demonstrated that higher levels of education improve self-rated health, while reducing loneliness and depression. Zhao and Dai (2022) proved that the higher the level of education received, the more it can inhibit excessive utilitarianism. Hou and Wang (2023) proved that the higher the degree of education, the stronger the subjective well-being, but it will reduce the individual well-being through the education mismatch effect and health damage effect. Wei (2023) proved that receiving higher education significantly improved the subjective well-being of individuals. Cheng et al. (2023) proved that the longer the years of education, the stronger the sense of happiness. Chen and Zhang (2023) proved that both education level and education return have a significant positive impact on the overall subjective well-being of residents. These views have proved that the longer the years of education, the stronger the subjective view of happiness. However, we also know that gender differences, age differences, urban and rural differences, marital differences, health differences, occupational differences, educational differences and so on all affect subjective well-being.

### 2.3 Women and subjective well-being

Donaghue (2009), proved that gender affects physical satisfaction and positive emotions and life satisfaction. van der Meer (2014) proved that work affects the subjective well-being of men and women. Women benefit from their partners’ work, while men do not. Wang et al. (2019) proved that subjective well-being can partially regulate the impact of marital relationship on women’s quality of life. Shenaar-Golan and Lans (2023) proved that women who did not have children were more satisfied with life and subjective well-being and had lower levels of emotional response. Ji and Zhao (2020) proved that family-based social capital indirectly improves women’s subjective well-being by reducing women’s psychological burden and reducing women’s depression. Wu (2020) proved that both absolute income and relative income have a positive impact on women’s subjective well-being. Huang and Zhang (2022) proved that the higher the frequency of physical exercise participation of female residents, the stronger the subjective well-being. Wang et al. (2023) proved that college brings higher returns to women than men, encourages parents to increase investment in their daughters’ education, and improves subjective well-being. Yu (2023) proved that subjective class identity has a significant positive impact on the well-being of female migrant workers, but there are intergenerational differences and regional differences. This shows that women’s health, women’s marriage and childbearing, women’s income, women’s physical exercise, women’s education, women’s subjective class identity and so on affect their subjective well-being.

We found that based on Mindsponge Theory, through a series of processing (Vuong, 2023), the existing literature focuses on the level of education, education promotion, years of education, higher education and other factors that affect subjective well-being, and also proposes that individuals and governments focus on improving the level of education. There are also literatures that focus on gender differences affecting subjective well-being. Women’s perception of happiness is more sensitive, and they pay attention to women’s development. However, there is a lack of special research on women’s years of education and subjective well-being. Whether it affects and how it affects provides the possibility for in-depth research. Based on this, this study proposes the following hypothesis:

*Hypothesis 1*. The longer the education years of women, the stronger the subjective well-being.

*Hypothesis 2*. Women receiving different years of education can have an impact on subjective well-being through economic status, physical and mental health, ecological environment, social cognition, and personal cognition.

## 3 Method

### 3.1 Data source

The data used in this study are from China general social survey (hereinafter referred to as CGSS). The CGSS project began in 2003 and is the earliest national, comprehensive and continuous academic survey project in China. CGSS survey data include economic, political, social, cultural and other aspects, and the research results are universal and authoritative. This study uses the latest 2021 CGSS data for analysis [hereinafter referred to as CGSS (2021)]. There are 8,148 original samples of CGSS (2021). According to the needs of this study, the samples in the original sample data that the respondents did not answer or answered invalid were removed and cleaned, and finally 4,469 research sample data were obtained. The final sample is universal and can reflect the relationship between women’s years of education and subjective well-being in China. The sample is representative.

### 3.2 Variable declaration

#### 3.2.1 Main variable

This study selects the degree of recognition of subjective well-being in CGSS (2021) to reflect the situation of subjective well-being, and selects subjective well-being as the main variable of this study. According to the question in CGSS (2021), “In general, do you feel happy in your life.” According to their own actual situation, the investigators choose “very unhappy,” relatively unhappy, “not happy,” “relatively happy,” “very happy,” and the corresponding values are: 1, 2, 3, 4, 5, 6. The larger the value, the stronger the subjective well-being.

#### 3.2.2 Explanatory variables

In this study, the years of education received by women are selected as the core explanatory variables. Based on CGSS (2021), “What is your current highest education level?” The results of the survey. In this study, the relevant parameters are assigned, as shown in Table 1. The larger the parameter assignment, the longer the years of education, the longer the years of education, the higher the level of education received.

**Table 1:** Explanatory variable related parameter assignment table.

Level of education	Time division of receiving education	Assignment
Did not receive any education	6 years or less	1
Home school, literacy class		
Primary school	6 years and 6 to 12 years	2
Junior high		
Vocational high school	12 years and 12 to 16 years	3
Ordinary high school		
Secondary technical school		
Technical school		
Junior college (adult higher education)		
College (formal higher education)		
Undergraduate (adult higher education)	16 years and 16 to 19 years	4
Undergraduate (formal higher education)		
Postgraduate and above	19 years and over 19 years	5

#### 3.2.3 Control variable

In this study, age, nationality, religious belief, marital status, fertility, rural household registration and housing situation were selected as the control variables of this study. For the “nation,” the Han nationality is assigned to 1, and the other nationalities are assigned to 2; for “religious belief,” the non-belief religion is assigned to 1, and the belief religion is assigned to 2; for “marital status,” the married is assigned a value of 1 and the unmarried is assigned a value of 2; for “fertility situation,” the fertility situation is assigned to 1, and the non-fertility situation is assigned to 2; for the “rural household registration situation,” the rural household registration is assigned to 1, and the non-rural household registration is assigned to 2; for the “housing situation,” the self-owned housing is assigned to 1, and the non-self-owned housing is assigned to 2.

#### 3.2.4 Mechanism variables

Based on Hypothesis 2, this study proposes that women receiving different degrees of education can affect subjective well-being through economic status, physical and mental health, ecological environment, social cognition, and personal cognition. Among them, according to the “economic situation” according to CGSS (2021), “In general, what is the income and expenditure of your family in the past year?”; aiming at the problem of “physical and mental health” according to the subjective well-being view in CGSS (2021), “I am very distressed about my health”; according to the “ecological environment in CGSS (2021), on the whole, do you think the environmental problems facing China are serious?”; aiming at the problem of “social cognition” according to the subjective well-being point of view in CGSS (2021), “I feel very confident in the development of society”; in view of the “personal cognition” according to the subjective well-being point of view in CGSS (2021), I am happy that my views have become more and more mature in recent years.

### 3.3 Rationale

#### 3.3.1 Ordered Logit model theory

Chen and Zhang (2023) used Probit model to study the influence of education level and return on education on residents’ subjective well-being. Wei (2023) used mixed regression method to study the effect and mechanism of higher education on individual subjective well-being. Cheng et al. (2023) constructed the model of education effort and happiness utility according to the principle of principal-agent general model, and used ordered Logit and OLS models to empirically analyze whether and how education plays a role in happiness. Wu (2023) conducted a quantitative regression analysis on the relationship between education level, subjective well-being and income of Chinese residents. For the study of such problems, scholars tend to use Logit model as the theoretical basis and method. For the Logit model, the function of the standard cumulative Logistic distribution is:


(1) $$F\left(x\right)=\frac{1}{1+e^{-x}}$$

Then, according to the relationship between Logit model and linear regression:


(2) $$L_{i}=\ln\left(\frac{P_{i}}{1-P_{i}}\right)=Z_{i}=\beta_{1}+\beta_{2}x_{i}$$

Finally, the above relationship is transformed into:


(3) $$\mathrm{Logit}\left(P_{i}\right)=\frac{1}{1+e^{-\left(\beta_{1}+\beta_{2}x_{i}\right)}}$$

Then the linear probability problem can be solved and estimated by adjusting and modifying according to the specific situation of the research. However, for the analysis of normal distribution function, the Probit model is better selected to establish the regression model. The Probit transformation and Probit regression model are as follows:


(4) $$\mathrm{Probit}\left(P\right)=\Phi^{-1}\left(P\right)=\beta_{0}+\beta_{1}x_{1}+\ldots +\beta_{n}x_{n}$$

The corresponding cumulative probability function, that is, the cumulative probability function of the standard normal distribution is:


(5) $$P=\Phi \left(\beta_{0}+\beta_{1}x_{1}+\ldots +\beta_{n}x_{n}\right)=\overset{\beta_{0}+\beta_{1}x_{1}+\cdots +\beta_{n}x_{n}}{\underset{-\infty }{\int }}\emptyset \left(z\right)dz$$

Therefore, after combining the above model theory, in order to explore the influence of education years on subjective well-being through economic status, physical and mental health, ecological environment, social cognition and personal cognition, the following models are established for analysis:


(6) $${\mathrm{happy}}_{i}=\mu +\alpha \times {\mathrm{educatyear}}_{i}+\overset{5}{\underset{i=0}{\sum }}\beta_{i}x_{1}+\delta_{i}$$

Among them, ${\mathrm{happy}}_{i}$ represents subjective well-being, and the larger the value, the stronger the subjective well-being; ${\mathrm{educatyear}}_{i}$ represents the years of education of female individuals. The higher the education level, the longer the years of education; $\overset{5}{\underset{i=0}{\sum }}\beta_{i}x_{1}$ represents the influence set of the five mechanism variables of economic status, physical and mental health, ecological environment, social cognition and personal cognition on subjective well-being; $\delta_{i}$ represents the error term.

#### 3.3.2 Coupling coordination degree model theory

The coupling coordination degree model is used to analyze the coordinated development level of things. The coupling degree refers to the correlation between two or more systems, which realizes the dynamic correlation of coordinated development, and can reflect the degree of interdependence and mutual restriction between systems. The degree of coordination refers to the degree of benign coupling in the coupling interaction relationship, which can reflect the quality of coordination.

In previous studies, few scholars have explored the factors between geographical regions in the study of women’s subjective well-being. However, there are great differences among different regions in China. In order to explore the relationship between women’s years of education and subjective well-being among different regions in China, this study uses the coupling coordination degree model to select Chongqing, Beijing, Hubei, Shandong, Zhejiang, Hunan, Fujian, Guangxi Zhuang Autonomous Region, Shaanxi and Gansu provinces and cities for analysis. Through the research and analysis of the coupling coordination degree model, we can further explore the deep relationship between the years of education and subjective well-being of women in different regions of China. This relationship will help policy makers to formulate different policies according to different regions to improve the subjective well-being of women in the region.

The model theory is as follows:

Coupling refers to the phenomenon that two or more systems or motion forms interact with each other through various interconnections. The coupling degree model of multiple systems is expressed as:


(7) $$C_{n}={\left\{\frac{u_{1}\cdot u_{2}\cdots u_{n}}{\prod \left(u_{1}+u_{2}\right)}\right\}}^{1/n}$$

In Equation 7, *C_n_* is the coupling degree of n-element system; *u*_1_…*u_n_* is the contribution of the first subsystem to the nth subsystem to the order degree of the total system, respectively. The calculation method is as follows:


(8) $$u_{i}=\overset{m}{\underset{i=1}{\sum }}w_{ij}u_{ij}$$

(9) $$\overset{m}{\underset{j=1}{\sum }}w_{ij}=1$$

In Equations 8, 9, *u_i_* are the contribution of the *i*th subsystem to the order degree of the total system; *u_ij_* is the normalized value of the *j*th index in the *i*th subsystem; *w_ij_* is the weight of the *j*th index in the *i*th subsystem, and the weight calculation of the index in each subsystem is calculated by the entropy weight method.

In some cases, the coupling degree index is difficult to reflect the “efficacy” and “synergy” effect of the subsystem as a whole. The upper and lower limits of the coupling degree of each subsystem index are taken from the extreme values of each index, and the extreme values are dynamic and unbalanced. It may be misleading to rely solely on the coupling degree discrimination. Therefore, the coupling coordination degree is proposed. Therefore, this study constructs a binary coupling coordination model based on women’s years of education and subjective well-being. The model is as follows:


(10) $$C=2{\left\{\frac{\left(\omega_{1}\cdot \omega_{2}\right)}{{\left(\omega_{1}+\omega_{2}\right)}^{2}}\right\}}^{1/2}$$

(11) $$D={\left(C\cdot T\right)}^{1/2}$$

(12) $$T=\left(m\omega_{1}+m\omega_{2}\right)$$

where *C* is the coupling degree, *D* is the coupling coordination degree, *ω*_1_ and *ω*_2_ represent the years of education and subjective well-being index of women respectively, *m* and *n* refer to the weight of years of education and subjective well-being index respectively, *T* is the coordination index value.

## 4 Results

### 4.1 Descriptive results analysis

**Figure 1:**
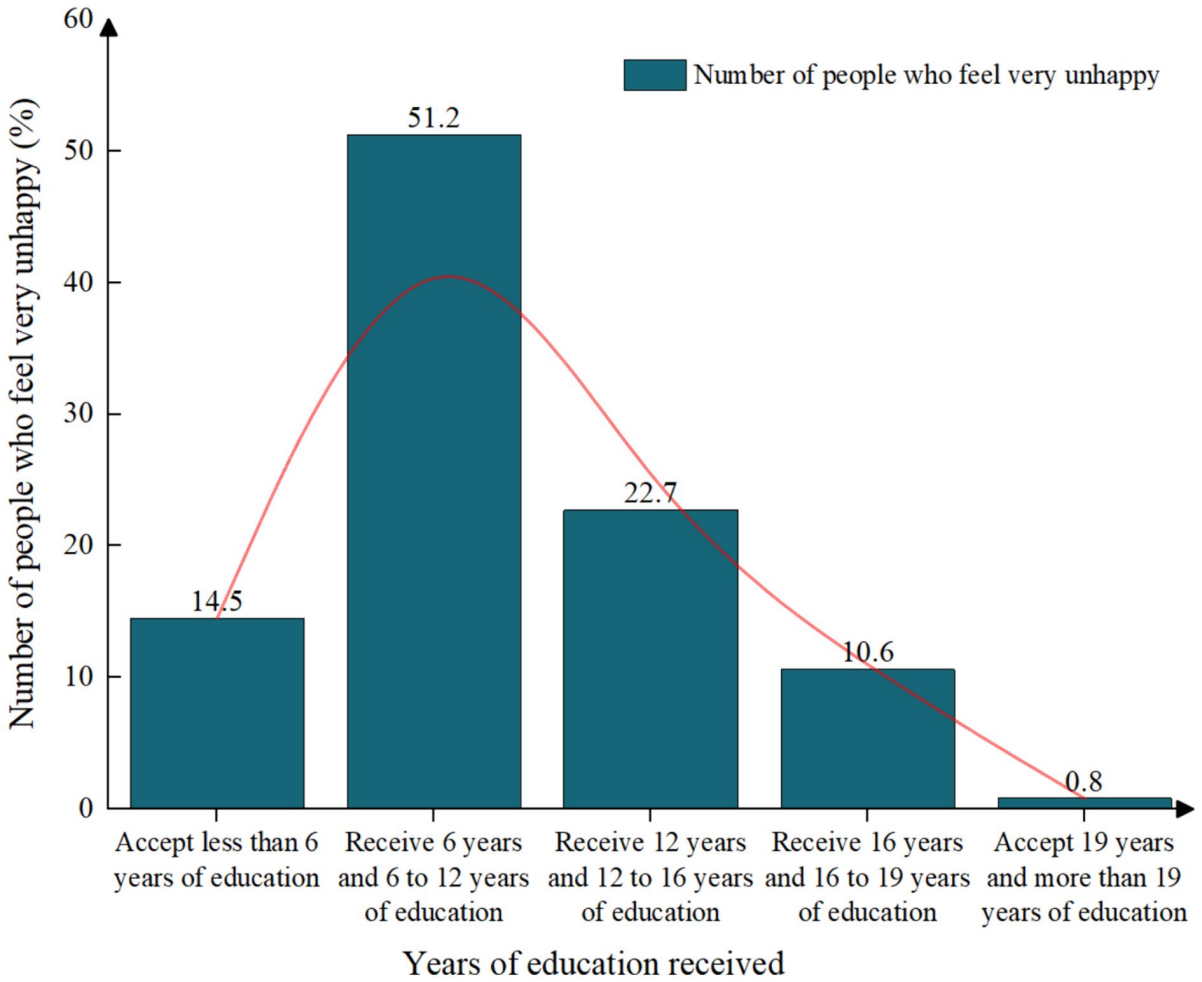
Distribution of women’s years of education and subjective unhappiness.

**Figure 2:**
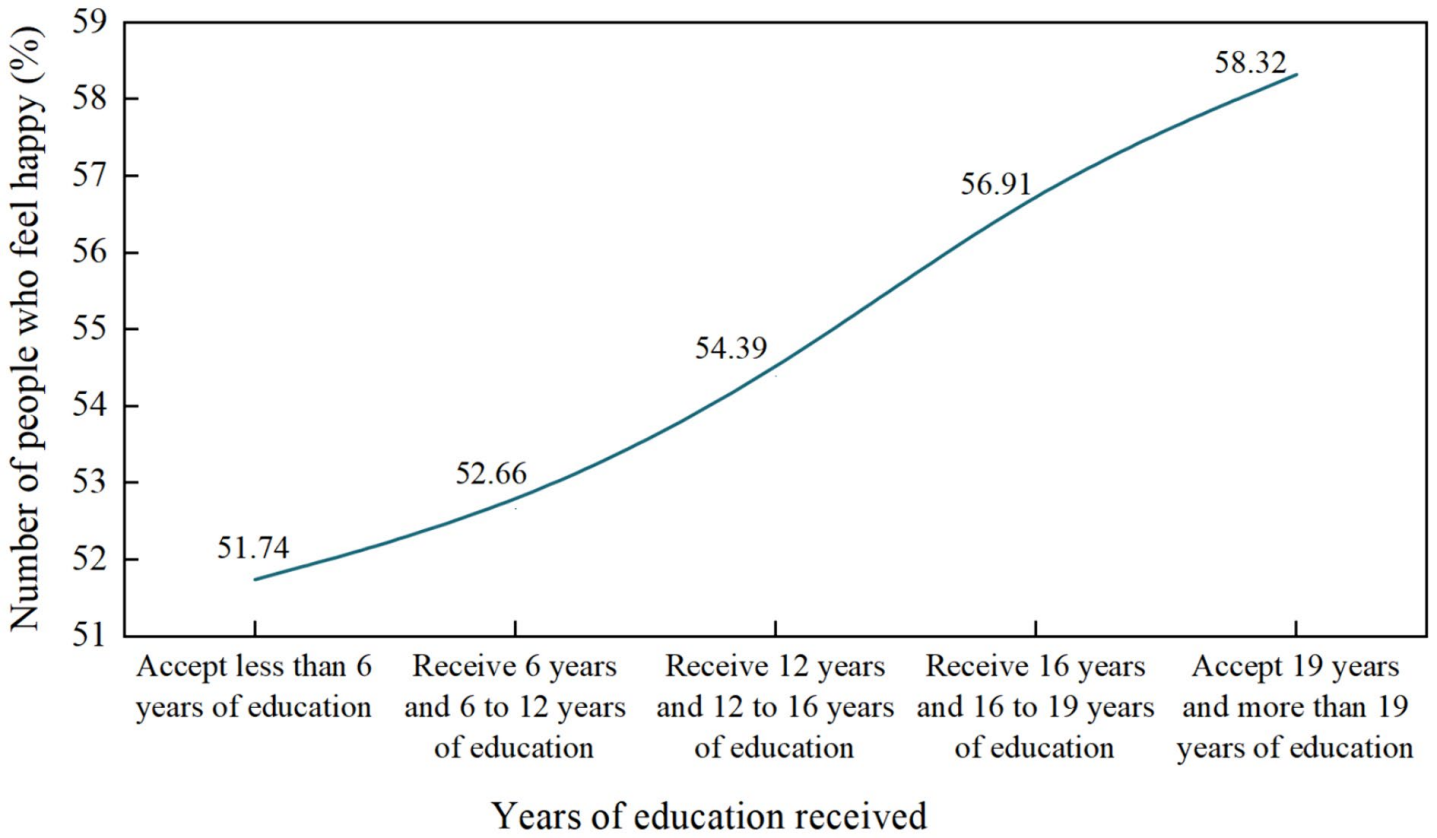
Trend chart of women’s years of education and feeling of happiness.

As shown in Table 2, the descriptive results of each variable comprehensively reflect the universality and comprehensiveness of the data samples in this study. As shown in Figure 1, we can find that with the increase of years of education, the proportion of women who feel very unhappy among the female group is gradually decreasing, which indicates that there is a corresponding correlation between years of education and women’s subjective well-being. As shown in Figure 2, we find that the proportion of women who feel happy is gradually increasing with the increase of years of education, which reflects that by receiving more education, women’s subjective well-being may be improved to a certain extent, which is completely consistent with the Hypothesis 1 of this study. At the same time, it also shows that the “women’s incompetence is virtue” in the traditional Chinese social concept is a wrong conceptual cognition. Education can not only improve women’s knowledge level, but also improve women’s subjective well-being. However, we should be concerned that some highly educated women may feel more pressure due to occupational stress, family conflicts or increased social expectations, resulting in decreased happiness.

**Table 2:** Variables descriptive statistics.

Variable	Observed value	Minimum value	Maximum value	Mean value	Standard deviation
Subjective well-being	4,469	1	5	3.18	1.322
Years of education	4,469	1	5	2.33	0.908
State of economy	4,469	1	2	1.08	0.268
Sound in body and mind	4,469	1	2	1.36	0.479
Ecological environment	4,469	1	2	1.08	0.264
Social cognition	4,469	1	2	1.08	0.268
Person cognition	4,469	1	2	1.13	0.332
Age	4,469	18	90	51.15	17.248
Nation	4,469	1	2	1.08	0.265
Religious belief	4,469	1	2	1.09	0.283
Marital status	4,469	1	2	1.29	0.453
Fertility condition	4,469	1	2	1.13	0.340
Rural Hukou	4,469	1	2	1.39	0.488
Housing conditions	4,469	1	2	1.50	0.500

### 4.2 Benchmark regression analysis

Based on the CGSS (2021) survey data, this study uses the ordered Logit model to perform regression analysis on Equation 6. Before the ordered Logit regression, the parallel line test is carried out on the research data, and the parallel line test is shown in Table 3.

**Table 3:** Parallel line test results.

Model	−2 log likelihood	Chi-square	Degree of freedom	Significance
Null hypothesis	442.765			
Routine	430.738	12.027	15	0.677

In the parallel line test table, the significance is 0.677, which is greater than 0.05, so the null hypothesis can be accepted. It is considered that the slope of each category of dependent variables is the same, and the parallel line test passes. Then, the overall fitting of the model is shown in Tables 4–6. As shown in Table 4, the significance in the model fitting information table is 0.000, so it can be considered that the overall fitting degree of the model is high.

**Table 4:** Model fitting information table.

Model	−2 log likelihood	Chi-square	Degree of freedom	Significance
Intercept	469.690			
Result	442.765	26.926	5	0.000

**Table 5:** Model goodness of fit table.

	Chi-square	Degree of freedom	Significance
Pearson	114.354	119	0.603
Deviation	119.405	119	0.472

**Table 6:** Pseudo R square table.

Cox-Snell	Negorko	McFadden
0.006	0.006	0.002

**Figure 3:**
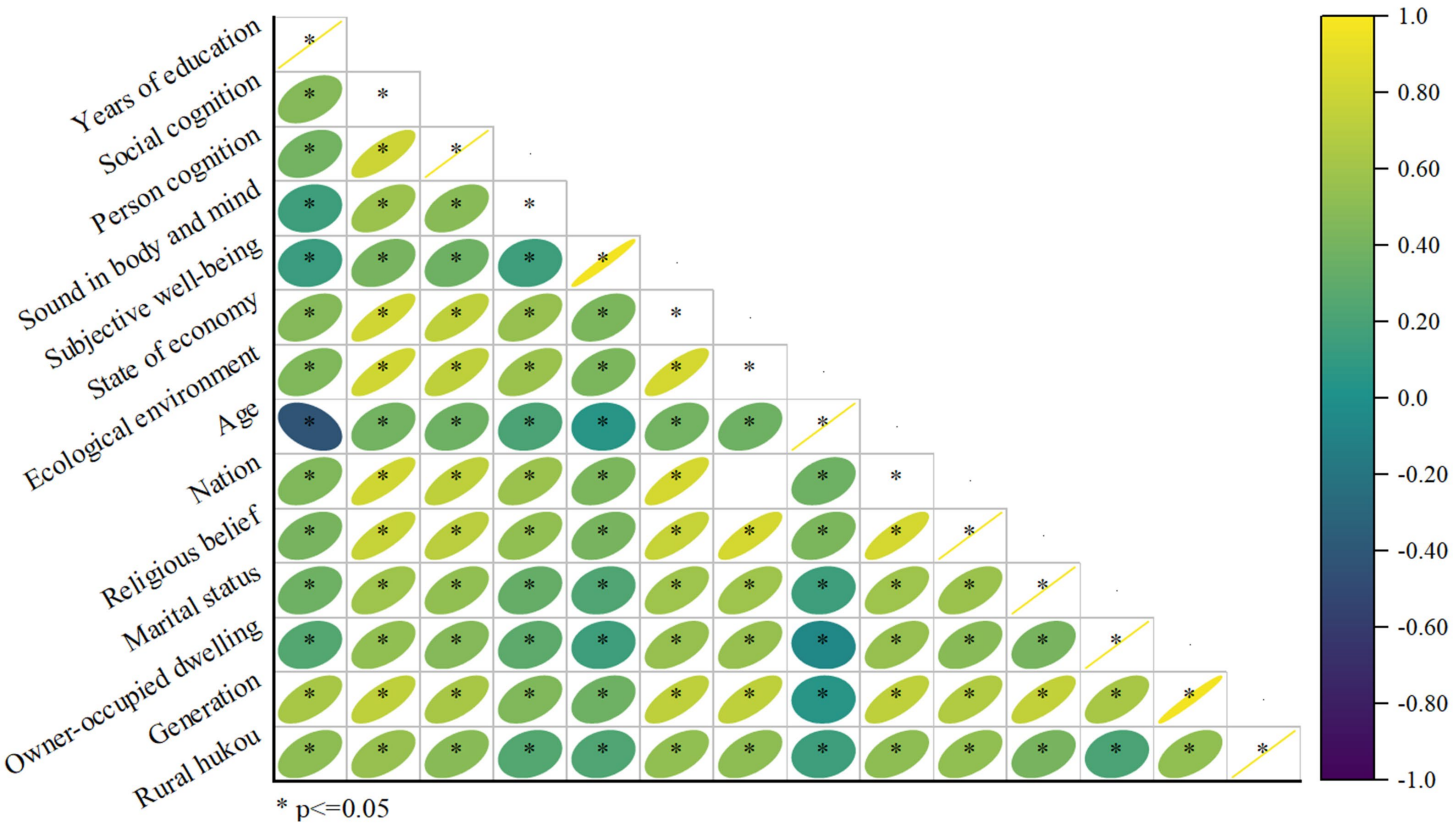
The influence of female education years on subjective well-being.

Finally, the relationship between the variables and subjective well-being is shown in Figure 3. At the same time, in the original Hypothesis 2, economic status, physical and mental health, ecological environment, social cognition, and personal cognition will have an impact on subjective well-being. After substitution into the model test, the ordered Logit regression results are shown in Table 7.

**Table 7:** Ordered Logit regression results.

	Regression coefficient	Standard error	Significant *p*-value	OR Value
State of economy	0.371	0.101	0.049**	1.449
Sound in body and mind	0.131	0.057	0.001***	1.156
Ecological environment	0.206	1.428	0.003***	1.237
Social cognition	0.272	0.103	0.000***	1.312
Person cognition	0.152	0.083	0.016**	1.162

***, **, * represents the significance level of 1, 5, 10% respectively.

According to the regression results, based on the economic status, physical and mental health, ecological environment, social cognition and personal cognition, the significant *p*-values are 0.049***, 0.001***, 0.003***, 0.000*** and 0.016** respectively, which are significant at the level, and the test results are in line with the research conclusions. Therefore, the above variables will have a significant impact on subjective well-being. We know that education, work, finance and health have a positive effect on individual subjective well-being (Ilies et al., 2019). In addition, our study shows in Table 7 that for every unit increase in economic status, the probability of subjective well-being increasing by one or more levels increases by 44.9%; for every unit increase in physical and mental health, the probability of subjective well-being increasing by one or more grades increased by 15.6%. For every unit increase in the ecological environment, the probability of subjective well-being increasing by one or more grades increased by 23.7%. For every unit increase in social cognition, the probability of subjective well-being increasing by one or more grades increased by 31.2%. For every unit increase in personal cognition, the probability of subjective well-being increasing by one or more grades increased by 16.2%.

Therefore, it can be proved that economic status, physical and mental health, ecological environment, social cognition and personal cognition will have an impact on subjective well-being, and will have a positive impact on improving subjective well-being, Hypothesis 2 is established.

### 4.3 Robustness test

To test the robustness of the results of this study. Firstly, some control variables are reduced. In the main regression, the control variables of age, nationality and religious belief are reduced and then regressed. If the results are significant, the robustness is proved. Secondly, this study re-adjusted the index assignment of subjective well-being, an main variable, to test the impact of different subjective well-being standards.

#### 4.3.1 Test for reducing control variables

The robustness of this study is shown in Table 8. Column (1) of Table 8 shows the significance of the effect of years of education and subjective well-being before the reduction of control variables. Table 8 (2) shows the significance of the effect of years of education and subjective well-being after the reduction of control variables. It can be observed that the significance has not changed before and after the reduction, so the results are robust.

**Table 8:** Robustness test results table.

	Subjective well-being	Subjective well-being	Subjective well-being	Subjective well-being
	(1)	(2)	(3)	(4)
Years of education	0.002*** (0.095)	0.006*** (0.088)	0.001*** (0.081)	0.000*** (0.028)
Constant term t	14.302	18.756	21.522	30.944
Regional fixed	Yes	Yes	Yes	Yes
Observed value	4,469	4,469	4,469	4,469
Goodness-of-fit	0.016	0.017	0.025	0.027
*R* ^2^	0.008	0.008	0.005	0.004

***, **, * represents the significance level of 1, 5, 10% respectively.

#### 4.3.2 Adjustment of the main variable

The way in which this study adjusts subjective well-being is to adjust the original “very unhappy” and “relatively unhappy” results to an assignment of 1, and to adjust “unhappy,” “relatively happy” and “very happy” to an assignment of 2. As shown in Table 8 (3), the results show that there is a significant impact on subjective well-being before adjustment. As shown in Table 8 (4), the results show that there is a significant impact on subjective well-being after adjustment. The results are significant, so the results are again proved to be robust.

### 4.4 Endogeneity test

This study mainly discusses the relationship between women’s years of education and subjective well-being. Combined with previous studies, it has been proved that higher education is associated with better mental health and personal subjective well-being (Lai et al., 2020; Li T. et al., 2023; Li X. et al., 2023). In this study, according to the study of the possible two-way causal relationship between women’s years of education and subjective well-being, the main variable years of education affect the main variable subjective well-being. At the same time, the explanatory variable subjective well-being also affects the explanatory variable years of education. Specifically, by increasing their years of education, women have a positive impact on subjective well-being from economic status, physical and mental health, ecological environment, social cognition and personal cognition; at the same time, women’s subjective well-being will also react to the years of education. In order to pursue their own subjective well-being, women will take the initiative to improve their years of education, so as to strengthen their acquisition of happiness.

To this end, this study selects the level of development in the region and the nature of female household registration as instrumental variables. First of all, the number of years of education has a certain relationship with the level of development and the nature of household registration in the region (Jin et al., 2020; Fang and Ye, 2022). The more developed the region, the longer the years of education; at the same time, women with urban household registration will generally receive more education than women with rural household registration, thus increasing the number of years of education. Secondly, this study uses the two-stage least squares (2SLS) method to estimate, separates the exogenous part, and conducts a new regression test. The results of endogenous test are shown in Table 9.

**Table 9:** Endogenous test results.

Variable	Years of education	Subjective well-being
Years of education		0.043*** (0.02)
The developed level of the region	0.020*** (0.005)	
Nature of household registration	0.041*** (0.009)	
Control variable	Yes	Yes
Observed value	4,469	4,469
Wald *F* statistic	299.209	—
*R* ^2^	0.653	0.186

***, **, * represents the significance level of 1, 5, 10% respectively.

Table 9 shows the results of one-stage and two-stage regression. The results of the first stage show that the level of development in the region and the nature of female household registration have positively affected the number of years of education for women, and the Wald *F* statistic value is 299.209 greater than 10, so there is no weak instrumental variable problem. The results of the second stage show that women’s years of education can significantly improve subjective well-being at the 1% significance level, indicating that women’s years of education are related to subjective well-being. The results are consistent with the findings of Qiu and Zhang’s (2021) research, indicating that the education level of women’s subjective happiness has a greater impact, and women’s education level should be continuously improved to achieve gender equality in education. In summary, the endogenous test results prove that the benchmark regression results of this study are effective.

### 4.5 Coupling coordination degree analysis

The above part uses the ordered Logit model to explore the relationship between women’s years of education and subjective well-being. After completing the above analysis, this study finds that the relationship between women’s years of education and subjective well-being in different regions of China is not consistent. In some areas, the increase in the number of years of education of female groups will actively promote their subjective well-being, but in other areas, the increase in the number of years of education of female groups will not significantly promote their subjective well-being. Therefore, by using the coupling coordination model, this study explores the coupling relationship between the years of education and subjective well-being of women in different regions of China, and explores the differences in the degree of influence of female groups in different regions, which is helpful for us to formulate different policies for the improvement of women’s subjective well-being in different regions of China.

This study selected Chongqing, Beijing, Hubei Province, Shandong Province, Zhejiang Province, Hunan Province, Fujian Province, Guangxi Zhuang Autonomous Region, Shaanxi Province, Gansu Province, the 10 provinces and cities for analysis. There are three reasons for the selection of these 10 provinces and cities: first, the geographical areas of these 10 provinces and cities are scattered, geographically distributed in the East, Central and West, and are representative; second, the economic development of these 10 provinces and cities has a hierarchy, both economically developed areas, such as Beijing, Zhejiang, etc., but also economically backward areas, such as Guangxi, with a hierarchy; the third is the distribution of population types. There are more female groups in these 10 provinces and cities, and the samples are in line with the theme of this study.

According to the analysis of the coupling coordination model, the results of the coupling coordination model are shown in Table 10.

**Table 10:** Coupling coordination degree results table.

Provinces, municipalities	Coordination index *T* value	Coupling coordination degree *D* value	Rank of harmony degree	Coupling coordination degree
Chongqing	0.598	0.7733045971672	8	Intermediate coordination
Beijing	0.892	0.9444575162494	10	Good coordination
Hubei province	0.304	0.5513619500836	6	Reluctant coordination
Shandong province	0.5	0.7071067811865	8	Intermediate coordination
Zhejiang province	0.794	0.8910667763978	9	Good coordination
Hu nan province	0.402	0.634034699365	7	Primary coordination
Fujian province	0.696	0.8342661445845	9	Good coordination
Gansu province	0.108	0.3286335345030	4	Mild disorders
Shaanxi province	0.206	0.4538722287164	5	On the verge of disorder
Guangxi Zhuang autonomous region	0.01	0.1	2	Serious imbalance

It can be seen from Table 10 that the degree of influence of women’s years of education and subjective well-being in different regions is different. Specifically, the degree of coupling coordination between women’s years of education and subjective well-being in Guangxi Zhuang Autonomous Region is the lowest, reaching the degree of serious imbalance, while the degree of coupling coordination between women’s years of education and subjective well-being in Beijing and Zhejiang Province is the highest, reaching the degree of quality coordination. This shows that in coastal areas and economically developed areas, the coupling between women’s years of education and subjective well-being is the strongest, and women can better achieve subjective well-being by improving their education level. In areas deviating from coastal areas and economically backward areas, the coupling relationship between women’s education level and subjective well-being is weak, and the degree of enhancing women’s subjective well-being by improving their education level is weak. Therefore, there are different differences between different regions. Policy makers in different regions need to formulate their own policies and regulations according to the actual situation of their own regions, so as to better improve the subjective well-being of women in the region.

## 5 Discussions

### 5.1 Women’s years of education have an impact on subjective well-being through different dimensions

The benchmark regression results show that women receiving different years of education can have an impact on subjective well-being through different dimensions such as economic status, physical and mental health, ecological environment, social cognition, and personal cognition. This reflects that women with longer years of education usually have higher incomes or better employment opportunities, are more likely to be guaranteed in physical and mental health, have more active actions on the ecological environment, and show criticality and independence in social participation and personal cognition, which has a positive effect on improving happiness. Among them, education level is related to subjective well-being, and the results are consistent with Cappa and Patton’s (2017). Health behavior has a beneficial effect on subjective well-being, and the results are consistent with the research of Stenlund et al. (2021). The impact of education on health and subjective well-being is consistent with the findings of Sheikh et al. (2017). However, it should be noted that different dimensions will not only have a positive impact on subjective well-being, but also have a negative impact. For example, some highly educated women may feel more pressure due to occupational stress, family conflict or increased social expectations, resulting in a decline in happiness. Secondly, highly educated women may face more social pressure and competition, which can lead to anxiety and social problems; finally, some highly educated women may bear greater psychological pressure in life. They have to pay more time in their careers and careers. Naturally, they spend less time in family business, and are more likely to feel the dual pressure of career and family.

### 5.2 The relationship between years of education and subjective well-being of women in different regions

The analysis of coupling coordination degree shows that the coordination degree of women’s years of education and subjective well-being in coastal areas, economically developed areas and urban areas is higher than that in inland areas, economically underdeveloped areas and rural areas. Education level and residence are related to subjective well-being, and the results are consistent with the research of Sun et al. (2018). Women in coastal areas, economically developed areas, and urban areas have economic conditions, rich educational resources, and abundant time. They can better achieve subjective well-being by improving their educational level. Women in inland areas, economically underdeveloped areas and rural areas are influenced by traditional concepts, have no family economic conditions to support them or lag behind in education level, and do not realize their subjective well-being by improving education level.

### 5.3 Limitations and future studies

#### 5.3.1 Limitations

First, our research focuses on the relationship and influencing factors between the years of education and subjective well-being of Chinese women. Whether the results are universal and whether they are applicable in other countries in the world need further verification. Second, our data come from the 2021 China General Social Survey. Although China’s comprehensive social survey data system is comprehensive and authoritative, the questionnaire design and research are not first-hand information. The research process needs to cooperate with and find source data. The initiative is not high, the in-depth research is not enough, and there are defects to a certain extent. Third, in our study, the measurement criteria for variables are relatively simple, mainly relying on a single question to assess the relationship between years of education and subjective well-being and its influencing factors. A single measurement method may not fully capture the complex relationship between years of education and subjective well-being, as well as the possible multi-dimensional influencing factors.

#### 5.3.2 Future studies

First, research methods and model construction can be innovated. For example, the system dynamics model is used to explore the synergistic relationship between women’s years of education and subjective well-being. Second, we can deeply study the influencing factors of marital relationship, family relationship and work relationship which are closely related to women’s living status, and how to achieve effective balance in these three complex relationships, so as to improve women’s years of education and better obtain happiness. Third, a more comprehensive and multi-dimensional scale can be used to measure the concept of subjective well-being. By designing a scale containing multiple related issues, we can more accurately reveal the internal relationship between years of education and subjective well-being, and provide richer and deeper data support. This approach will help to improve the accuracy and effectiveness of research and provide a more reliable basis for research in the field of women’s subjective well-being.

## 6 Conclusion

In summary, the relationship between women’s years of education and subjective well-being is complex and diverse, including both positive and negative aspects. Education can improve women’s happiness, but it may also be accompanied by some negative effects, especially in a highly competitive and stressful environment. Based on the above research results, this study puts forward the following three suggestions:

First, government departments should pay more attention to the formulation and implementation of education policies. Let women, ethnic minority residents, disabled people get more educational opportunities, cognitive social skills, in order to face the comprehensive pressure of individual, family and social development, so as to obtain subjective well-being.

Second, vigorously develop the social economy and improve the living standards of residents. Let the society be more dynamic, let the culture be pluralistic and inclusive, and make life colorful. In an equal social environment, women get longer education years, more social interaction, less anxiety and more relaxation, so as to improve their subjective well-being.

Third, balance the family relationship between husband and wife, children and mother-in-law. Husbands need to be more responsible for children’s education and housework. Mother-in-law need to rationally look at the difference between the concept of “parenting” and the traditional concept of “filial piety.” Give more support and understanding to each other to improve personal subjective well-being.

## Data Availability

Publicly available datasets were analyzed in this study. This data can be found at: Chinese General Social Survey: http://cgss.ruc.edu.cn/.
